# Assessment of self-esteem among Tunisian cannabis users

**DOI:** 10.1192/j.eurpsy.2024.838

**Published:** 2024-08-27

**Authors:** M. Kacem, W. Bouali, M. Henia, S. Brahim, L. Zarrouk

**Affiliations:** Psychiatric department, Taher Sfar Hospital of Mahdia, Mahdia, Tunisia

## Abstract

**Introduction:**

Self-esteem plays a role in the adaptive functioning of the human being. It could be a protective factor regarding multiple risks particularly substance use.

**Objectives:**

The aim of this study is to assess self-esteem among a group of young Tunisian users of cannabis.

**Methods:**

The total study sample was composed of 137 participants, who took part of a transversal descriptive study during two months (January and February 2020). These partakers were comprehensively recruited among Emergency patients of Mahdia Hospital. Thereupon, the main criteria for the selection of these patients was their consumption of cannabis, regardless of their primary health care seeking reason or purpose. The research was carried out upon their declaration of cannabis consumption and their compliance to be part of such a study. Thus, Data were collected on a pre-determined data sheet that included various information (age, sex, lifestyle, personal and family psychiatric history, age at which they started their cannabis consumption and the rate of cannabis use …). Accordingly, Self-esteem was assessed using the Rosenberg Self-Esteem Scale (RSES). Consequently, the interview took place after the subject’s verbal and informed consent and the assurance of anonymity and confidentiality of the interview content.

**Results:**

In our study population, the cannabis consumers were young adults aged between 18 and 35 years old, with a male predominance of 71%. Among those users, 65.9% were single and 29.7% dropped out of school or experienced academic failure. On a socio-economic level, we concluded to a rate of 5.8% (lower class), 60.9% (middle class) and 33.3% (upper class). Besides, 40.8% were employed. In total, 23.2% had a psychiatric history. Furthermore, the use of other substances was also prominent and frequent as follows: alcohol 72.5%, tobacco 74.6%, ecstasy 41.3% and 25.4% cocaine. The use of cannabis was considered as a means of indulgence and pleasure for 66.7%, as an anxiolytic for 26.8% and as a sedative for 23.9%. Self-esteem, among those cannabis users, was very low in 20% of cases, low in 38% of cases, medium in 15% of cases and high in 25% of cases. Consequently, more than half of the study population remains below the medium average according to RSES.

**Conclusions:**

These results lead us to question the relation between cannabis and self-esteem. The question that is evolved about the use of cannabis is the following: Is it used as a remedy or is it the cause of self-esteem deficiency?

**Disclosure of Interest:**

None Declared

